# Relationships between physical activity and other health-related measures using state-based prevalence estimates

**DOI:** 10.34172/hpp.2023.36

**Published:** 2023-12-16

**Authors:** Peter D. Hart, Nestor Asiamah, Getu Teferi, Ivan Uher

**Affiliations:** ^1^Glenville State University, Glenville, WV 26351, USA; ^2^Health Promotion Research, Havre, Montana, USA; ^3^Kinesmetrics Lab, Tallahassee, Florida, USA; ^4^School of Health and Social Care, University of Essex, Colchester, UK; ^5^Department of Sports Science, Debremarkos University, Debremarkos, Ethiopia; ^6^Institute of Physical Education and Sport, Pavol Jozef Šafárik University, 040 01 Košice, Slovakia

**Keywords:** Physical activity, Muscle stretching exercises, Obesity, Health behavior, Behavioral risk factor surveillance system

## Abstract

**Background:**

Both physical activity and muscle-strengthening activity have known relationships with other health-related variables such as alcohol and tobacco use, diet, and health-related quality of life (HRQOL). The purpose of this study was to explore and quantify the associations between physical activity measures and health-related variables at the higher state level.

**Methods:**

This cross-sectional study used data from the 2017 and 2019 Behavioral Risk Factor Surveillance System surveys. State-based prevalence (%) estimates were computed for meeting physical activity guidelines (PA), meeting muscle-strengthening activity guidelines (MS), both PA and MS (MB), drinking alcohol (D1), heavy alcohol drinking (HD), fruit consumption (F1), vegetable consumption (V1), good self-rated health (GH), overweight (OW), obesity (OB), current smoking (SN), and smokeless tobacco use (SL). Descriptive statistics, correlation coefficients, and data visualization methods were employed.

**Results:**

Strongest associations were seen between PA and F1 (2017: *r*=0.717 & 2019: *r*=0.695), MS and OB (2017: *r*=-0.781 & 2019: *r*=-0.599), PA and GH (2017: *r*=0.631 & 2019: *r*=0.649), PA and OB (2017: *r*=-0.645 & 2019: *r*=-0.763), and MB and SN (2017: *r*=-0.713 & 2019: *r*=-0.645). V1 was associated only with PA (2017: *r*=0.335 & 2019: *r*=0.357) whereas OW was not associated only with PA. Canonical correlation analysis showed the physical activity variables were directly related (*r*_ c_=0.884, *P*<0.001) to the health variables.

**Conclusion:**

This study used high-level data to support the many known relationships between PA measures and health-related variables.

## Introduction

 Physical activity (PA) is a known preventive health behavior with increased amounts associated with decreased health risk and thus promoted to all adult populations.^1^ Accordingly, *Healthy People 2030* has an objective to increase the percent of adults 18 + years of age to 52.9% (from 47.9% in 2020) who engage in aerobic PA of at least moderate intensity for 150 + minutes per week, or 75 + minutes per week of vigorous intensity, or an equivalent combination.^2^ Muscle strengthening activity (MSA) is a specific form of PA and is also promoted to adults as a preventive health behavior.^3^ Another *Healthy People 2030* objective, related to MSA, is to increase the percent of adults 18 + years of age to 36.6% (from 31.9% in 2020) who perform muscle-strengthening activity on 2 + days per week.^4^ Both PA and MSA are promoted in combination to adult populations and can serve as an additional PA guideline measure for individuals meeting both recommendations.^5^

 The influence that PA has on health outcomes can be direct, indirect, or both in nature.^6^ For example, PA can directly improve a person’s cardiovascular disease risk by reducing blood pressure.^7^ Similarly, MSA can directly improve an individual’s functional ability by improving their muscular strength and balance.^8^ PA can also contribute to positive health outcomes indirectly by first influencing a different health behavior or outcome. For example, PA can indirectly improve a person’s cancer risk by stimulating the desire to improve nutrition and adhere to the American Cancer Society (ACS) Guideline for Diet and Physical Activity.^9^ In this scenario, the ACS diet would be directly related to improved cancer risk.

 There is a large body of knowledge published on the health-related correlates of PA that directly relate to health outcomes in adult populations. For instance, objectively measured PA has been shown proportionally related to other health behaviors like smoking, alcohol use, and diet quality.^10-12^ PA has consistently been associated with different measures of perceived health, such as health-related quality of life (HRQOL), general health, and life satisfaction.^13-15^ Research also supports MSA as a positive predictor of other health-related behaviors directly associated with health outcomes.^16-18^

 Evidence of these aforementioned associations between PA measures and health-related behaviors is important for both preventive medicine and individual-level behavior change. But a more comprehensive understanding of these relationships can be achieved when examining them additionally at a higher level of analysis. That is to say, if prevalence estimates of certain behaviors are collected across varying geographic locations, such as U.S. states, these estimates can serve as correlational data from a higher observational level. One such study from the Centers for Disease Control and Prevention (CDC) reported this type of analysis by correlating alcohol-use behaviors among youth with those of adults, across U.S. states.^19^ Another study used state-based prevalence of obesity to find their associations with six different types of cancer using data from a national health survey and the U.S. Cancer Statistics.^20^

 Considering this, examining the extent to which state-based PA estimates influence other health measures can be an important contribution to population health. The aim of this study was to explore and quantify the associations between PA measures and other health-related variables at a higher observational level using state-based prevalence estimates.

## Materials and Methods

###  Study procedures

 A cross-sectional correlational design was employed to address the study’s research question. Data came from the 2017 and 2019 U.S. CDC’s Behavioral Risk Factor Surveillance System (BRFSS) surveys. Details regarding the BRFSS background and design can be found elsewhere.^21,22^ Briefly, the BRFSS is a state-based annual telephone survey designed to collect consistent data on health-risk behaviors, health conditions, and preventive care in noninstitutionalized U.S. adults 18 + years of age. In this analysis, data from all available states were included without the use of the District of Columbia, Guam, and Puerto Rico. This resulted in *N* = 50 state records for the 2017 BRFSS and *N* = 49 state records for the 2019 BRFSS (No data for New Jersey). The 2017 and 2019 BRFSS surveys were the most recent, to date, assessing MSA.

###  PA measures and health-related variables

 Twelve different health variables were used in this study, each representing a state’s weighted prevalence (%) estimate.^23^ Aerobic PA represents the prevalence of adults that participate in 150 minutes or more of aerobic PA per week. Muscle strengthening exercise (MS) represents the prevalence of adults that participate in muscle strengthening exercises two or more times per week. Meeting both PA and MS (MB) represents the prevalence of adults that participated in enough aerobic and muscle strengthening exercises to meet guidelines (i.e., PA and MS). Drinking alcohol (D1) represents the prevalence of adults who have had at least one drink of alcohol within the past 30 days. Heavy drinking (HD) represents the prevalence of heavy alcohol consumption as defined as an adult male having more than 14 drinks per week or an adult female having more than 7 drinks per week. Fruit consumption (F1) represents the prevalence of consuming fruit one or more times per day. Vegetable consumption (V1) represents the prevalence of consuming vegetables one or more times per day. General health (GH) represents the prevalence of self-rated good health or better. Obese (OB) represents the prevalence of obesity as assessed by a body mass index (BMI) within the 30.0 kg/m^2^ to 99.8 kg/m^2^ range. Overweight (OW) represents the prevalence of overweight as assessed by a BMI within the 25.0 kg/m^2^ to 29.9 kg/m^2^ range. Smoking (SN) represents the prevalence of adults who are current smokers. Smokeless tobacco use (SL) represents the prevalence of adults who currently use chewing tobacco, snuff, or snus every day.

###  Statistical analyses

 Data analysis for this study included (1) descriptive statistics, (2) normality and outlier analyses, (3) Pearson correlations for bivariate associations, (4) data visualization methods for displaying prevalence estimates and correlations with 95% confidence intervals (CIs), and (5) canonical correlation analysis on the set of PA measures and set of other health variables. The purpose of the normality and outlier analyses was to further explore the state-based prevalence data as well as to check for major violations of Pearson correlation coefficient assumptions. The correlation analysis was replicated using Spearman correlations and each coefficient was found to be similar in direction and magnitude and therefore not presented. Data visualization techniques included (a) a prevalence plot by state using average 2017 and 2019 values (see description below), (b) bivariate scatter plots for each year (i.e., 2017 and 2019) for each PA measure and health variable pair, with fit line, (c) Forest plots of the Pearson correlations with their 95% CI, by year, for each PA measure (i.e., PA, MS, and MB), (d) canonical correlation analysis path diagram, representing two constructs of PA and health, and (e) scatter plot with fit regression line for the first canonical PA (i.e., x variable) and health (i.e., y variable) variates.

 The canonical correlation analysis also used average 2017 and 2019 prevalence data with an *N* = 50. Since the SL prevalence was missing for Rhode Island in 2017, its 2019 value was used as the average value. Additionally, since New Jersey did not have reliable data in 2019, their 2017 prevalence estimates were used as average values. Multicollinearity was checked during the canonical correlation analysis and MB was dropped from the set of PA variables due to high variance inflation (i.e., *VIF* > 10). JMP version 16 (SAS Institute Inc., Cary, NC), SAS version 9.4 (SAS Institute Inc., Cary, NC), and R version 4.2 (R Foundation for Statistical Computing, Vienna, Austria) (ggplot and DiagrammeR) were used for all analyses.^24-26^

## Results

**Figure 1:**
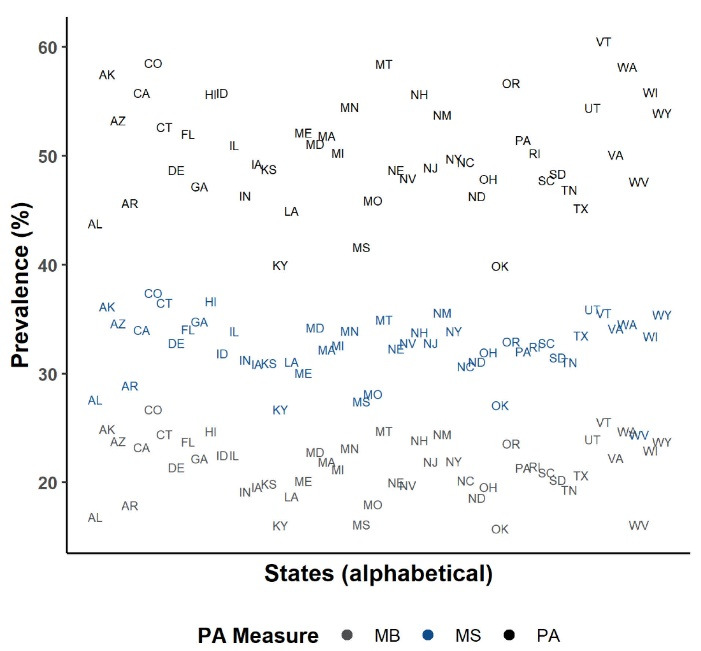
Plot of physical activity (PA) prevalence by physical activity (PA) measure for each state, Behavioral Risk Factor Surveillance System (BRFSS) 2017 and 2019

Table 1 contains descriptive statistics, normality checks, and outlier analysis for the 2017 BRFSS prevalence estimates with a total sample size of *N* = 50 for all variables, except SL due to the lack of Rhode Island data on that variable. It is clear that on average the prevalence of PA (Mean = 50.6%, SD = 4.5%) is greater than MS (Mean = 30.1%%, SD = 2.7%) and MB (Mean = 20.2%, SD = 2.6%). Table 2 contains the same exploratory data analyses but for the 2019 BRFSS prevalence estimates with a total sample size of N = 49 for all variables (minus New Jersey). Similarly, the average prevalence of PA (Mean = 50.6%, SD = 5.6%) is greater than MS (Mean = 34.9%%, SD = 3.5%) and MB (Mean = 22.6%, SD = 3.3%). Figure 1 displays these findings visually with 2017 and 2019 prevalence estimates averaged.

**Table 1:** Descriptive and normality statistics for state-based health-related prevalence estimates, Behavioral Risk Factor Surveillance System (BRFSS) 2017

**Variable**	**Mean**	**Median**	**SD**	**Min**	**Max**	**Skew**	**Z**_Skew_	**Kurt**	**Z**_Kurt_	**Outliers**	***P***
PA	50.58	50.40	4.53	41.90	59.70	0.15	0.44	-0.68	-0.97	0	> 0.150
MS	30.13	30.20	2.74	22.70	35.50	-0.27	-0.77	0.09	0.12	1	> 0.150
MB	20.19	20.20	2.59	15.10	26.00	0.03	0.10	-0.75	-1.08	0	> 0.150
D1	54.02	54.90	7.44	30.80	65.80	-0.93	-2.66	0.91	1.30	1	> 0.150
HD	6.35	6.30	1.16	3.60	8.90	-0.11	-0.31	0.19	0.28	0	> 0.150
F1	63.38	63.30	4.31	53.70	70.40	-0.66	-1.89	-0.17	-0.24	0	0.014
V1	81.90	81.95	2.38	76.10	87.60	0.09	0.26	0.21	0.31	0	> 0.150
GH	81.94	82.35	3.28	74.10	87.30	-0.71	-2.03	-0.05	-0.07	0	> 0.150
OB	30.75	31.45	3.73	22.60	38.10	-0.09	-0.27	-0.64	-0.92	0	> 0.150
OW	35.26	35.30	1.20	32.60	39.00	0.15	0.42	0.98	1.41	1	> 0.150
SN	17.34	17.15	3.50	8.90	26.00	0.28	0.81	0.05	0.08	0	> 0.150
SL	2.49	2.20	1.40	0.60	6.10	0.71	2.04	0.13	0.18	1	> 0.150

Note. *N* = 50. (*N* = 49 for SL). All states participated in 2017. Descriptive statistics are for state-based prevalence estimates (%s). *SD* is standard deviation. Skew is skewness. Kurt is kurtosis. Z_Skew_ and Z_Kurt_ are the Z statistics for skewness (skew/sqrt(6/N)) and kurtosis (kurt/sqrt(24/N)), respectively. Outliers is the number of % values with a standard score greater than |2.5|. *P *value is for the Kolmogorov-Smirnov normality test. PA: Participated in 150 minutes or more of aerobic physical activity per week. MS: Participated in muscle strengthening exercises two or more times per week. MB: Participated in enough aerobic and muscle strengthening exercises to meet guidelines. D1: Adults who have had at least one drink of alcohol within the past 30 days. HD: Heavy drinkers (adult men having more than 14 drinks per week and adult women having more than 7 drinks per week). F1: Consumed fruit one or more times per day. V1: Consumed vegetables one or more times per day. GH: Good or better health. OB: Obese (BMI 30.0 kg/m^2^ – 99.8 kg/m^2^). OW: Overweight (BMI 25.0 kg/m^2^– 29.9 kg/m^2^). SN: Adults who are current smokers. SL: Adults who currently use chewing tobacco, snuff, or snus every day.

**Table 2:** Descriptive and normality statistics for state-based health-related prevalence estimates, Behavioral Risk Factor Surveillance System (BRFSS) 2019

**Variable**	**Mean**	**Median**	**SD**	**Min**	**Max**	**Skew**	**Z**_Skew_	**Kurt**	**Z**_Kurt_	**Outliers**	***P***
PA	50.58	49.90	5.63	35.30	62.30	-0.40	-1.14	0.63	0.89	1	> 0.150
MS	34.93	35.50	3.50	26.10	39.70	-0.84	-2.39	-0.01	-0.01	1	> 0.150
MB	22.60	23.00	3.28	15.30	28.50	-0.47	-1.34	-0.24	-0.34	0	> 0.150
D1	52.97	54.00	7.37	31.10	64.60	-0.81	-2.31	0.57	0.82	1	> 0.150
HD	6.65	6.40	1.21	4.20	9.40	0.57	1.64	0.07	0.11	0	< 0.010
F1	60.15	60.60	4.08	51.60	68.20	-0.25	-0.72	-0.61	-0.87	0	> 0.150
V1	79.96	79.70	2.78	74.50	87.50	0.64	1.82	0.81	1.16	1	> 0.150
GH	81.87	81.80	3.22	73.40	86.30	-0.59	-1.69	-0.22	-0.31	1	> 0.150
OB	32.19	32.30	3.85	23.80	40.80	-0.16	-0.46	-0.39	-0.56	0	> 0.150
OW	34.83	34.60	1.27	31.90	37.90	0.18	0.50	0.30	0.43	0	> 0.150
SN	16.33	16.00	3.29	7.90	23.80	0.06	0.18	0.20	0.29	1	> 0.150
SL	2.54	2.30	1.46	0.40	6.30	0.71	2.03	-0.15	-0.21	1	> 0.150

Note. *N* = 49. All states participated in 2019 less NJ. Descriptive statistics are for state-based prevalence estimates (%s). *SD* is standard deviation. Skew is skewness. Kurt is kurtosis. Z_Skew_ and Z_Kurt_ are the Z statistics for skewness (skew/sqrt(6/N)) and kurtosis (kurt/sqrt(24/N)), respectively. Outliers is the number of % values with a standard score greater than |2.5|. p-value is for the Kolmogorov-Smirnov normality test. PA: Participated in 150 minutes or more of aerobic physical activity per week. MS: Participated in muscle strengthening exercises two or more times per week. MB: Participated in enough aerobic and muscle strengthening exercises to meet guidelines. D1: Adults who have had at least one drink of alcohol within the past 30 days. HD: Heavy drinkers (adult men having more than 14 drinks per week and adult women having more than 7 drinks per week). F1: Consumed fruit one or more times per day. V1: Consumed vegetables one or more times per day. GH: Good or better health. OB: Obese (BMI 30.0 kg/m^2^ – 99.8 kg/m^2^). OW: Overweight (BMI 25.0 kg/m^2^– 29.9 kg/m^2^). SN: Adults who are current smokers. SL: Adults who currently use chewing tobacco, snuff, or snus every day.

 The normality and outlier analyses was judged overall as acceptable for both years because (1) the few number of outliers, (2) the relatively small skewness and kurtosis statistics, (3) the rejected normality test for only one variable (F1 in 2017 and HD in 2019), (4) the subjective opinion that the histograms looked appropriate, (5) all prevalence data were checked and found to be accurate, and (6) Spearman correlations were not judged different from the Pearson correlation coefficients in the upcoming bivariate analyses.

**Figure 2:**
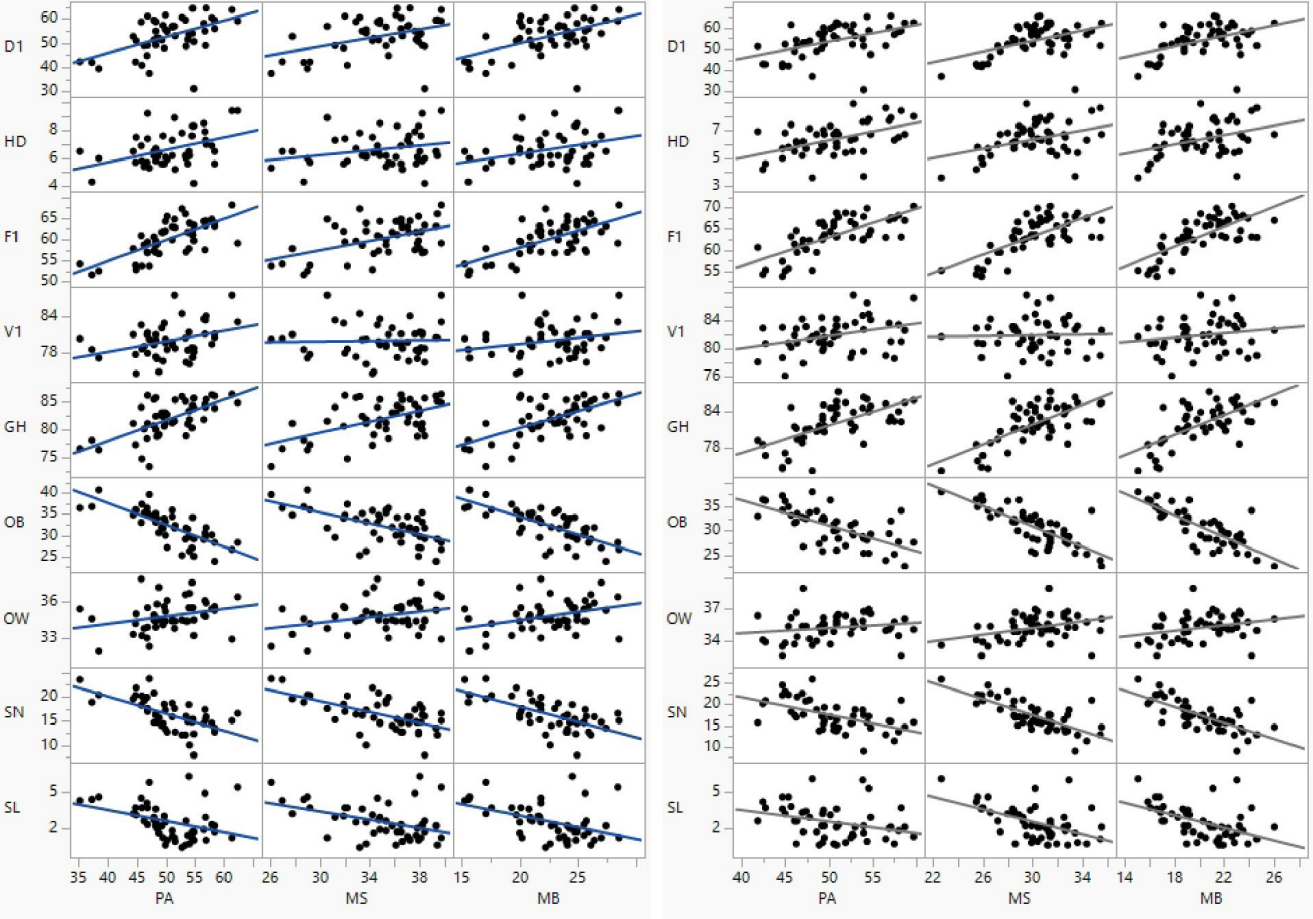
Scatter plots for physical activity (PA) measure and health variable prevalence estimates with fit linear regression lines for Behavioral Risk Factor Surveillance System (BRFSS) 2017 (left) and 2019 (right)

**Figure 3:**
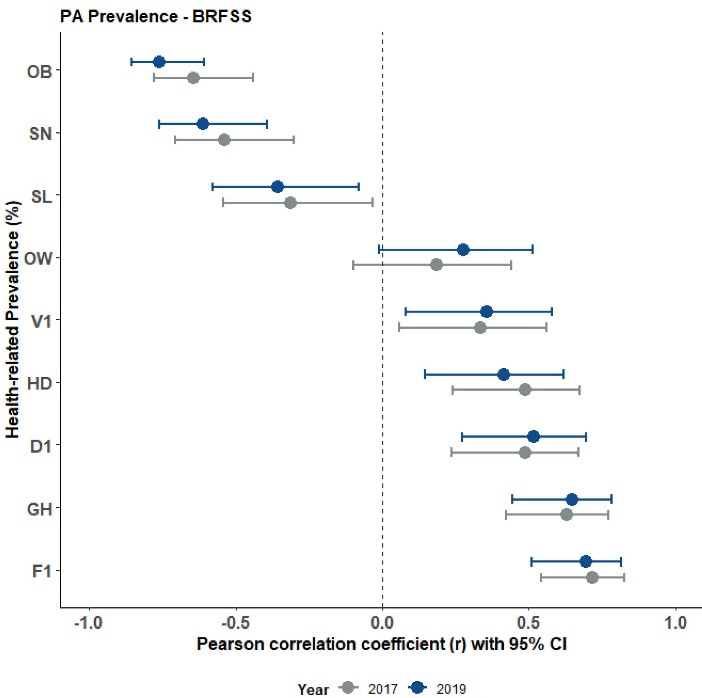
Forest plot of Pearson correlations for meeting physical activity guidelines (PA) and health-related variables, Behavioral Risk Factor Surveillance System (BRFSS) 2017 and 2019

**Figure 4:**
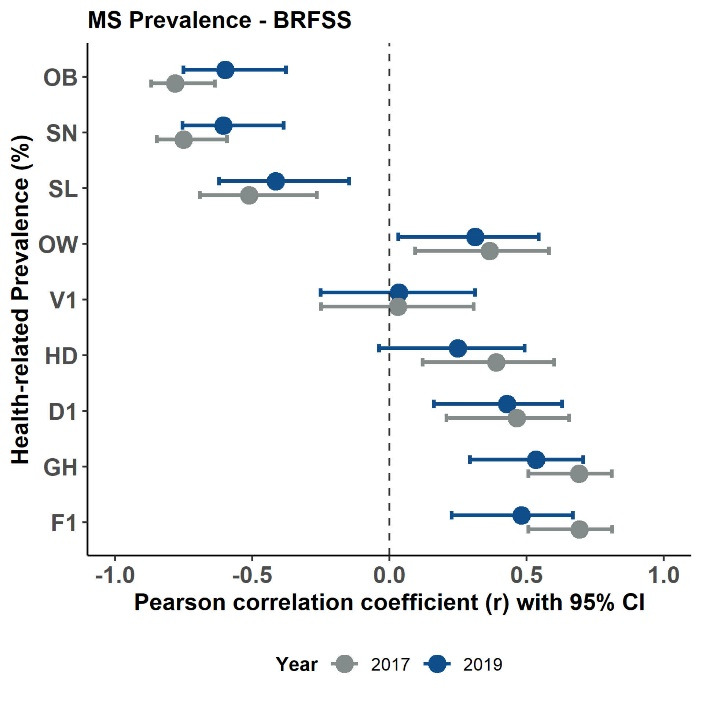
Forest plot of Pearson correlations for meeting muscle-strengthening activity guidelines (MS) and health-related variables, Behavioral Risk Factor Surveillance System (BRFSS) 2017 and 2019

**Figure 5:**
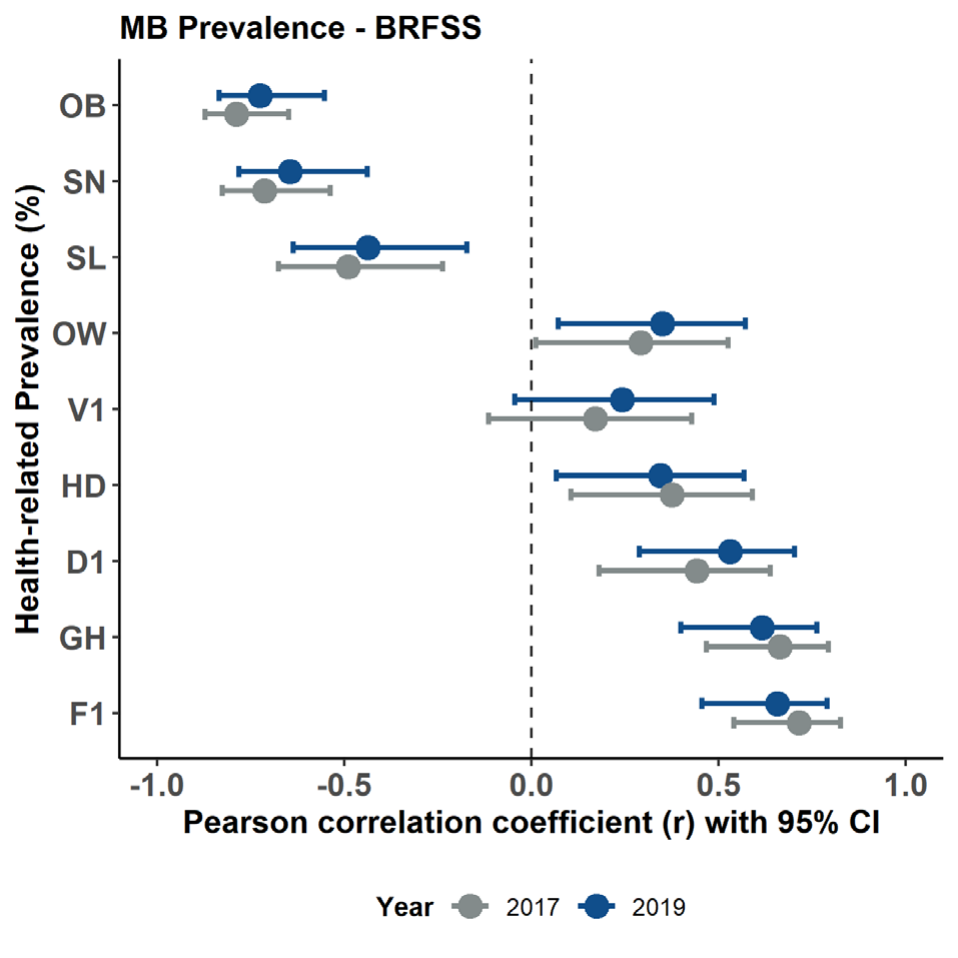
Forest plot of Pearson correlations for meeting both physical activity and muscle-strengthening activity guidelines (MB) and health-related variables, Behavioral Risk Factor Surveillance System (BRFSS) 2017 and 2019

Table 3 contains the bivariate Pearson correlation coefficients for the PA measure and health variable prevalence estimates for the 2017 BRFSS data. Table 4 contains the same bivariate correlations for the 2019 BRFSS data. Figure 2 displays the scatter plots for these relationships with fit linear regression lines - confirming the assumption of linearity for the correlations. Collectively, most state-based correlations were significant (*P* < 0.05) with strongest associations seen between PA and F1 (2017: *r* = 0.717 & 2019: *r* = 0.695), PA and OB (2017: *r* = -0.645 & 2019: *r* = -0.763), MS and OB (2017: *r* = -0.781 & 2019: *r* = -0.599), PA and GH (2017: *r* = 0.631 & 2019: *r* = 0.649), and MB and SN (2017: *r* = -0.713 & 2019: *r* = -0.645). V1 was associated only with PA (2017: *r* = 0.335 & 2019: *r* = 0.357) whereas OW was not associated only with PA. Associations were consistent between years less MS and HD (2017: *r* = 0.390, *P* = 0.005 & 2019: *r* = 0.249, *P* = 0.085). Figures 3, 4 and 5 display these correlations along with their 95% CIs.

**Table 3:** Pearson correlation coefficients for state-based health-related prevalence estimates, Behavioral Risk Factor Surveillance System (BRFSS) 2017

**Variable**	**D1**	**HD**	**F1**	**V1**	**GH**	**OB**	**OW**	**SN**	**SL**	**Average**
PA	0.487	0.488	0.717	0.335	0.631	-0.645	0.186	-0.540	-0.313	0.482
*P*	< 0.001	< 0.001	< 0.001	0.017	< 0.001	< 0.001	0.197	< 0.001	0.028	
MS	0.464	0.390	0.692	0.031	0.691	-0.781	0.366	-0.750	-0.512	0.520
*P*	0.001	0.005	< 0.001	0.831	< 0.001	< 0.001	0.009	< 0.001	< 0.001	
MB	0.441	0.376	0.716	0.171	0.664	-0.788	0.292	-0.713	-0.490	0.517
*P*	0.001	0.007	< 0.001	0.234	< 0.001	< 0.001	0.040	< 0.001	< 0.001	
Average	0.464	0.418	0.708	0.179	0.662	0.738	0.281	0.668	0.439	

Note. *N* = 50. (*N* = 49 for SL). All states participated in 2017. Average is the mean of the absolute value of correlation coefficients. PA: Participated in 150 minutes or more of aerobic physical activity per week. MS: Participated in muscle strengthening exercises two or more times per week. MB: Participated in enough aerobic and muscle strengthening exercises to meet guidelines. D1: Adults who have had at least one drink of alcohol within the past 30 days. HD: Heavy drinkers (adult men having more than 14 drinks per week and adult women having more than 7 drinks per week). F1: Consumed fruit one or more times per day. V1: Consumed vegetables one or more times per day. GH: Good or better health. OB: Obese (BMI 30.0 kg/m^2^ – 99.8 kg/m^2^). OW: Overweight (BMI 25.0 kg/m^2^– 29.9 kg/m^2^). SN: Adults who are current smokers. SL: Adults who currently use chewing tobacco, snuff, or snus every day.

**Table 4:** Pearson correlation coefficients for state-based health-related prevalence estimates, Behavioral Risk Factor Surveillance System (BRFSS) 2019

**Variable**	**D1**	**HD**	**F1**	**V1**	**GH**	**OB**	**OW**	**SN**	**SL**	**Average**
PA	0.517	0.414	0.695	0.357	0.649	-0.763	0.275	-0.613	-0.358	0.516
*P*	< 0.001	0.003	< 0.001	0.012	< 0.001	< 0.001	0.056	< 0.001	0.012	
MS	0.428	0.249	0.482	0.034	0.535	-0.599	0.313	-0.605	-0.415	0.407
*P*	0.002	0.085	0.001	0.819	< 0.001	< 0.001	0.029	< 0.001	0.003	
MB	0.531	0.345	0.658	0.242	0.616	-0.726	0.349	-0.645	-0.436	0.505
*P*	< 0.001	0.015	< 0.001	0.094	< 0.001	< 0.001	0.014	< 0.001	0.002	
Average	0.492	0.336	0.611	0.211	0.600	0.696	0.312	0.621	0.403	

Note. *N* = 49. All states participated in 2019 less NJ. Average is the mean of the absolute value of correlation coefficients. PA: Participated in 150 minutes or more of aerobic physical activity per week. MS: Participated in muscle strengthening exercises two or more times per week. MB: Participated in enough aerobic and muscle strengthening exercises to meet guidelines. D1: Adults who have had at least one drink of alcohol within the past 30 days. HD: Heavy drinkers (adult men having more than 14 drinks per week and adult women having more than 7 drinks per week). F1: Consumed fruit one or more times per day. V1: Consumed vegetables one or more times per day. GH: Good or better health. OB: Obese (BMI 30.0 kg/m^2^ - 99.8 kg/m^2^). OW: Overweight (BMI 25.0 kg/m^2^- 29.9 kg/m^2^). SN: Adults who are current smokers. SL: Adults who currently use chewing tobacco, snuff, or snus every day.

**Figure 6:**
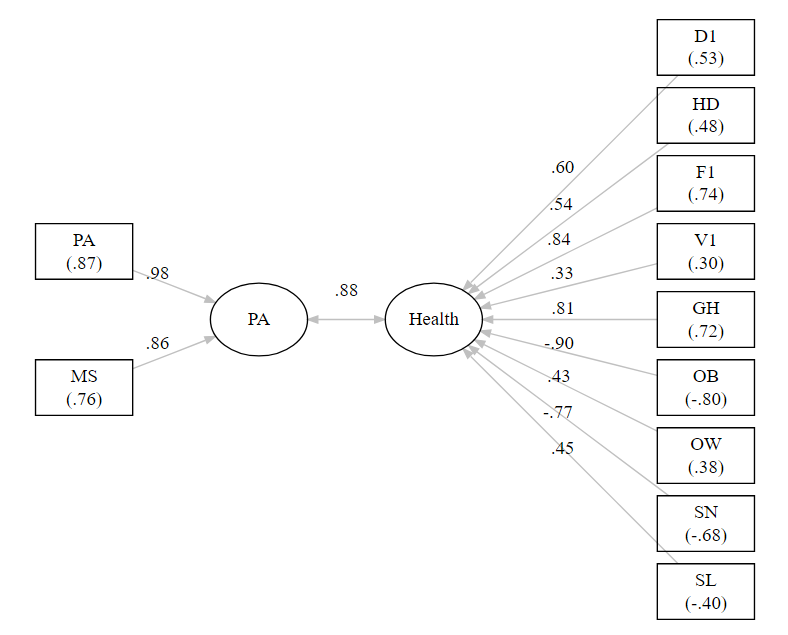
Canonical correlation analysis path diagram highlighting the overall correlation between physical activity (PA) and health and displaying within construct (cross construct) loadings, Behavioral Risk Factor Surveillance System (BRFSS) 2017 and 2019.

**Figure 7:**
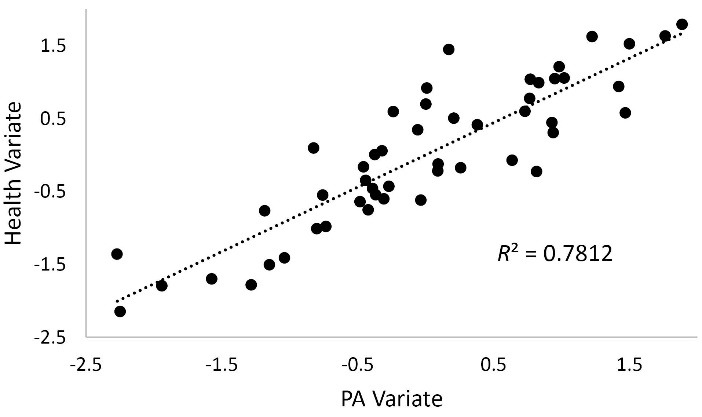
Scatter and fit line for the first canonical variate scores for physical activity (PA) and health, Behavioral Risk Factor Surveillance System (BRFSS) 2017 and 2019

Table 5 displays results of the canonical correlation analysis using PA and MS measures as one set of PA variables and D1, HD, F1, V1, GH, OB, OW, SN, and SL as a set of health variables. These results show the linear combination of PA variables are strongly related (*r*_c_ = 0.884, *P* < 0.001) to the linear combination of health variables. Figure 6 displays the canonical correlation analysis path diagram highlighting the overall correlation between PA and health. Examining the graph path loadings, it is clear that both PA (*r*_s.w_ = 0.983) and MS (*r*_s.w_ = 0.861) strongly correlate with PA and OB (*r*_s.w_ = -0.899), F1 (*r*_s.w_ = 0.842), SN (*r*_s.w_ = -0.765), and GH (*r*_s.w_ = 0.814) strongly with health. Examination of the between (cross) construct loadings show that both PA (*r*_s.b_ = 0.868) and MS (*r*_s.b_ = 0.761) strongly correlate with health and OB (*r*_s.b_ = -0.795), F1 (*r*_s.b_ = 0.744), SN (*r*_s.b_ = -0.676), and GH (*r*_s.b_ = 0.720) strongly with PA. Figure 7 displays the scatter and fit line for the first canonical scores with large explained variance (*R*^2^ = 0.78).

**Table 5:** Canonical correlation analysis of physical activity (PA) and health prevalence, Behavioral Risk Factor Surveillance System (BRFSS) 2017 and 2019

**Constructs**	**First variate**			
	**b**	**B**	***r***_s.w_	***r***_s.b_
PA variables				
PA	0.160	0.771	0.983	0.868
MS	0.101	0.282	0.861	0.761
RI (%)	66.7			
Health variables				
D1	-0.008	-0.062	0.603	0.533
HD	0.255	0.286	0.538	0.475
F1	0.157	0.650	0.842	0.744
V1	-0.067	-0.167	0.334	0.295
GH	0.014	0.044	0.814	0.720
OB	-0.146	-0.544	-0.899	-0.795
OW	-0.163	-0.171	0.434	0.384
SN	-0.053	-0.178	-0.765	-0.676
SL	0.303	0.431	-0.452	-0.399
RI (%)	34.0			
*r*_c_	0.884			
%	81.2			
*F, p*	8.18, < 0.001			
λ, *p*	0.120, < 0.001			

*Note*. *b* is raw canonical coefficients (within construct slopes). *B* is standardized canonical coefficients (within construct standardized slopes). *r*_s.w_ is within construct structure loadings (correlations). *r*_s.b_is between (cross) construct structure loadings (correlations). RI is the Stewart-Love redundancy index. *r*_c_ is canonical correlation coefficient. % represents proportion of the sum of eigenvalues explained by the first canonical variate. *F* statistic is for likelihood ratio test and λ is Wilks’ Lambda with null hypothesis stating the canonical correlation is zero.The 2nd canonical variates was also significant but with low explained variance and uninterpretable.

## Discussion

 This study used a novel approach for examining associations between PA measures and health-related variables. Specifically, state-level prevalence (%) estimates, weighted and representing the U.S. civilian adult population, were used for bivariate correlation data. Findings showed that most state-level health-related variables were associated with all three state-level PA measures. Predictably, the prevalence of OB was negatively correlated strongly (all *r* < -0.60) with all three PA measures. The prevalence of OW, however, was not correlated with PA and only moderately positively correlated with MS and MB. These findings are noteworthy since individual level associations appear mixed regarding PA and overweightness.^27,28^ Most other findings were consistent with known evidence at the individual level, such as positive correlations between PA measures and perceived health (GH), fruit consumption (F1), alcohol use (D1), heavy drinking (HD) and negative correlations between PA measures and SN and smokeless tobacco use (SL).^29-34^ An exception was the weak associations between PA measures and vegetable consumption (V1), with just a modest correlation between V1 and PA and no relationship between V1 and MS or MB.^35^

 This study also used a novel multivariate statistical technique, canonical correlation analysis, to examine the extent to which the set of PA measures (i.e., PA and MS) correlate with the set of health-related variables (i.e., D1, HD, F1, V1, GH, OB, OW, SN, and SL). the results clearly indicated a strong association (*r*_c_ = 0.884) between the two sets of health variables. This may be the only application, to date, where such a multivariate correlation coefficient has been computed using U.S. state-level prevalence estimates of health variables.

 A strength regarding this study is its use of BRFSS data and its use of representative samples of noninstitutionalized adults for estimation of health-related summary statistics. Another strength regarding this study is the series of survey questions and modules assessing various health risk behavior and health status outcomes. The BRFSS specifically designs its questionnaires to target the leading causes of premature death and disability in the U.S. Therefore, the variables used in this study are of the utmost importance to the health status of U.S. adults.

 There are limitations worth mentioning. Firstly, this study uses a higher level of analysis unit, in the form of state-based prevalence estimates, and can be considered ecological data. Therefore, findings from this study should not necessarily imply that the same associations exist at the individual level (i.e., ecological fallacy).^36^ Secondly, BRFSS data are cross-sectional in nature and thus do not provide evidence for cause-and-effect. Specifically, these findings are not implying that changing a person’s PA will subsequently change their health behavior or health status. Thirdly, all variables in this study were assessed via self-report telephone interviews. Therefore, participant misclassification cannot be ruled out due to measurement and reporting bias. In sum, findings from this study should be considered with caution.

## Conclusion

 This study used higher-level data to support the many known relationships between PA and health. Findings clearly showed moderately strong associations between the different PA measures and F1, GH, OB, and SN among U.S. adults. Conversely, findings showed consistently weak associations between the PA measures and V1 and OW. Thus, at the state level, PA may provide little information regarding adult overweightness status and vegetable consumption. State-based associations between PA and health can be an alternative source of needs assessment for health promotion professionals and policy makers.

## Acknowledgments

 We thank the volunteers who participated in the 2017 and 2019 Behavioral Risk Factor Surveillance System.

## Competing Interests

 The authors declare that they have no competing interests.

## Ethical Approval

 This study used data freely accessible and downloadable, in public domain, at the U.S. Centers for Disease Control and Prevention (CDC). Therefore, institutional review board approval was not required.

## References

[1] U.S. Department of Health and Human Services. Physical Activity Guidelines for Americans. 2nd ed. 2018. Available from: https://health.gov/paguidelines/second-edition/pdf/Physical_Activity_Guidelines_2nd_edition.pdf. Accessed April 10, 2023.

[2] Office of Disease Prevention and Health Promotion. Physical Activity. Healthy People 2030. U.S. Department of Health and Human Services. Available from: https://health.gov/healthypeople/objectives-and-data/browse-objectives/physical-activity/increase-proportion-adults-who-do-enough-aerobic-physical-activity-substantial-health-benefits-pa-02. Accessed April 10, 2023.

[3] Piercy KL, Troiano RP, Ballard RM, Carlson SA, Fulton JE, Galuska DA (2018). The physical activity guidelines for Americans. JAMA.

[4] Office of Disease Prevention and Health Promotion. Physical Activity. Healthy People 2030. U.S. Department of Health and Human Services. Available from: https://health.gov/healthypeople/objectives-and-data/browse-objectives/physical-activity/increase-proportion-adults-who-do-enough-muscle-strengthening-activity-pa-04. Accessed April 10, 2023.

[5] Hart PD (2021). Relationship between meeting physical activity guideline parameters and body mass index (BMI) in adults. J Phys Act Res.

[6] Blair SN, Jacobs DR Jr, Powell KE (1985). Relationships between exercise or physical activity and other health behaviors. Public Health Rep.

[7] Joseph G, Marott JL, Torp-Pedersen C, Biering-Sørensen T, Nielsen G, Christensen AE (2019). Dose-response association between level of physical activity and mortality in normal, elevated, and high blood pressure. Hypertension.

[8] Andreu-Caravaca L, Ramos-Campo DJ, Chung LH, Martínez-Rodríguez A, Rubio-Arias J (2023). Effects and optimal dosage of resistance training on strength, functional capacity, balance, general health perception, and fatigue in people with multiple sclerosis: a systematic review and meta-analysis. Disabil Rehabil.

[9] Rock CL, Thomson C, Gansler T, Gapstur SM, McCullough ML, Patel AV (2020). American Cancer Society guideline for diet and physical activity for cancer prevention. CA Cancer J Clin.

[10] Domazet SL, Tarp J, Thomsen RW, Højlund K, Stidsen JV, Brønd JC (2022). Accelerometer-derived physical activity and sedentary behaviors in individuals with newly diagnosed type 2 diabetes: a cross-sectional study from the Danish nationwide DD2 cohort. Front Sports Act Living.

[11] Dodge T, Clarke P, Dwan R (2017). The relationship between physical activity and alcohol use among adults in the United States. Am J Health Promot.

[12] Xu F, Cohen SA, Lofgren IE, Greene GW, Delmonico MJ, Greaney ML (2018). Relationship between diet quality, physical activity and health-related quality of life in older adults: findings from 2007-2014 National Health and Nutrition Examination Survey. J Nutr Health Aging.

[13] Alzahrani H (2022). Dose-response association between physical activity and health-related quality of life in general population: a population-based pooled study. Healthcare (Basel).

[14] Hart PD, Benavidez G, Erickson J (2017). Meeting recommended levels of physical activity in relation to preventive health behavior and health status among adults. J Prev Med Public Health.

[15] Alonzo R, Lalva T, Couper RG, Wilk P (2022). Association between physical activity and life satisfaction among adults with multimorbidity in Canada. Can J Public Health.

[16] Hart PD (2019). Grip strength and health-related quality of life in US adult males. J Lifestyle Med.

[17] Hart PD, Buck DJ (2019). The effect of resistance training on health-related quality of life in older adults: systematic review and meta-analysis. Health Promot Perspect.

[18] Bennie JA, De Cocker K, Teychenne MJ, Brown WJ, Biddle SJH (2019). The epidemiology of aerobic physical activity and muscle-strengthening activity guideline adherence among 383,928 US adults. Int J Behav Nutr Phys Act.

[19] Nelson DE, Naimi TS, Brewer RD, Nelson HA (2009). State alcohol-use estimates among youth and adults, 1993-2005. Am J Prev Med.

[20] Kumar S, Moghaddam S, Goldberg DS, Mantero A, Crane TE (2022). Increasing rates of pancreatic and hepatocellular cancers parallel increasing obesity rates. Pancreas.

[21] Centers for Disease Control and Prevention (CDC). Behavioral Risk Factor Surveillance System Overview: BRFSS 2017. CDC; 2018.

[22] Centers for Disease Control and Prevention (CDC). Behavioral Risk Factor Surveillance System Overview: BRFSS 2019. CDC; 2019.

[23] Centers for Disease Control and Prevention (CDC). Behavioral Risk Factor Surveillance System (BRFSS): Complex Sampling Weights and Preparing 2019 BRFSS Module Data for Analysis. CDC; 2020.

[24] JMP®, Version 16. Cary, NC: SAS Institute Inc; 1989-2023.

[25] SAS Institute Inc. SAS/STAT® 14.1 User’s Guide. Cary, NC: SAS Institute Inc; 2015.

[26] R Core Team. R: A Language and Environment for Statistical Computing. Vienna, Austria: R Foundation for Statistical Computing; 2022. Available from: https://www.R-project.org/.

[27] Hart PD (2022). Bivariate and multivariate associations between physical activity and body measure variables in US adults, 2017-2020 pre-pandemic. J Phys Act Res.

[28] Dickerson JB, Smith ML, Benden ME, Ory MG (2011). The association of physical activity, sedentary behaviors, and body mass index classification in a cross-sectional analysis: are the effects homogenous?. BMC Public Health.

[29] Kim J, Im JS, Choi YH (2017). Objectively measured sedentary behavior and moderate-to-vigorous physical activity on the health-related quality of life in US adults: the National Health and Nutrition Examination Survey 2003-2006. Qual Life Res.

[30] Cohen A, Ardern CI, Baker J (2017). Physical activity mediates the relationship between fruit and vegetable consumption and cognitive functioning: a cross-sectional analysis. J Public Health (Oxf).

[31] Courtney JB, Russell MA, Conroy DE (2022). Tobacco and cannabis use as moderators of the association between physical activity and alcohol use across the adult lifespan in the United States: NHANES, 2005-2016. Prev Med.

[32] Dodge T, Clarke P (2018). Testing weight motives and guilt/shame as mediators of the relationship between alcohol use and physical activity. Addict Behav.

[33] Hart PD (2021). Relationship between health risk behaviors and physical inactivity in Montana adults. J Phys Act Res.

[34] Gutema BT, Chuka A, Ayele G, Estifaons W, Melketsedik ZA, Tariku EZ (2021). Tobacco use and associated factors among adults reside in Arba Minch health and demographic surveillance site, southern Ethiopia: a cross-sectional study. BMC Public Health.

[35] Loprinzi PD (2016). Association between accelerometer-determined physical activity and flavonoid-rich fruit and vegetable consumption among a national sample of US adults. Prev Med Rep.

[36] Idrovo AJ (2011). Three criteria for ecological fallacy. Environ Health Perspect.

